# A case of congenital ureteral atresia causing rare upper and lower urinary tract manifestations in a puppy: a case report

**DOI:** 10.1186/s12917-021-02780-6

**Published:** 2021-02-09

**Authors:** Megan Zalek, Rohan Shah, Timothy Bolton

**Affiliations:** 1 Department of Biomedical and Diagnostic Sciences, University of Tennessee College of Veterinary Medicine, University of Tennessee, Tennessee Knoxville, USA; 2 Dogs and Cats Veterinary Referral and Emergency Hospital, Maryland Bowie, USA; 3 Department of Small Animal Clinical Sciences, Virginia-Maryland College of Veterinary Medicine, Virginia Tech University, Virginia Blacksburg, USA

**Keywords:** Ureteral atresia, Hydronephrosis, Hydroureter, Lower urinary tract signs, Dog

## Abstract

**Background:**

Ureteral atresia is the congenital absence of a ureteral opening, resulting in a blind-ended ureter that fails to terminate at the urinary bladder. Consequently, severe hydroureter and hydronephrosis occur ipsilateral to the atresic ureter. However, hydronephrosis contralateral to severe hydroureter, although reported in humans, is not documented in the dog. Additionally, ureteral atresia has not been reported as a cause for lower urinary tract signs directly related to extramural urinary bladder compression. This report aims to describe these unique manifestations of this congenital urinary tract disease, as well as follow-up findings after successful treatment.

**Case presentation:**

A 4-month-old male Husky puppy was evaluated for pollakiuria, stranguria, and urine dribbling of 1-month duration. During the physical examination, a mass was palpated in the mid-abdomen. Diagnostic imaging and cystoscopy findings were diagnostic for right-sided ureteral atresia with secondary hydroureter and hydronephrosis. The severe right hydroureter caused lower urinary tract signs and contralateral hydronephrosis secondary to regional compression of the left distal ureter and urinary bladder. A right-sided ureteronephrectomy was performed, resolving the stranguria and pollakiuria. Significant reduction in the contralateral (left) hydronephrosis also occurred.

**Clinical Relevance:**

Ureteral atresia should be considered as a differential diagnosis for lower urinary tract signs and/or bilateral hydronephrosis in a young dog. Reporting this case expands our knowledge of congenital lower urinary tract disease and the etiology of their manifestations in dogs. Surgical resolution of the congenital ureteral abnormality can result in preservation of renal function in the contralaterally obstructed kidney.

## Background

Congenital ureteral anatomic anomalies causing obstructive hydroureter and hydronephrosis are rare in veterinary small animal patients, with various etiologies reported [1–10]. Ureteral atresia, one such anomaly, is the absence of a ureteral opening that results in a blind-ended ureter failing to terminate at the urinary bladder [11]. Hydroureter, hydronephrosis, and a loss in function of the ipsilateral kidney occur secondary to the obstruction. Only one case of ureteral atresia has been recorded in the veterinary literature to date [1].

In humans, hydroureter is a reported cause of contralateral ureteral obstruction and bilateral hydronephrosis; however, to the authors’ knowledge, this has not been reported in the dog [12, 13]. Hydroureter due to ureteral atresia has also not been reported to cause lower urinary tract signs due to extramural urinary bladder compression. In the present case study, we describe both bilateral hydronephrosis and lower urinary tract signs related to ureteral atresia-induced hydroureter in a puppy. We also describe appropriate treatment and follow up for this condition.

## Case presentation

A 4-month-old, male intact Siberian Husky weighing 11.8 kg was evaluated because of a 1-month history of stranguria, pollakiuria, and urine dribbling. One week prior to presentation, the referring veterinarian prescribed enrofloxacin^1^ 5.8 mg/kg PO q24h for treatment of a lower urinary tract infection diagnosed after finding isosthenuria (specific gravity 1.012) and leukocytes on free-catch dipstick urinalysis. Despite this therapy, the lower urinary tract signs persisted and the dog was referred for evaluation (visit #1).

**Fig. 1:**
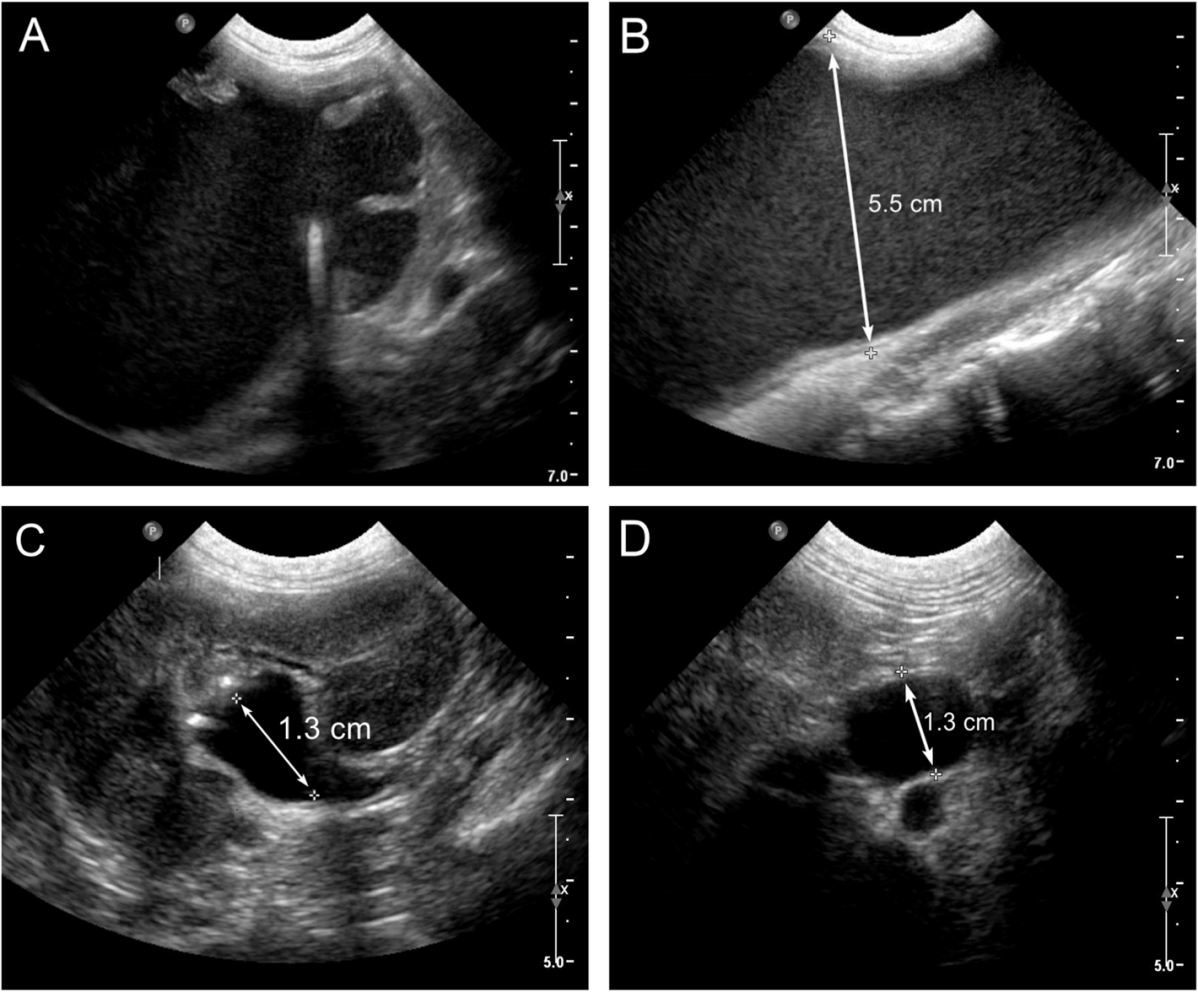
Abdominal ultrasound findings for a dog with distal ureteral atresia. **a** Sagittal view of the right kidney showing a complete loss of renal architecture. **b** Transverse view of the right ureter showing severe dilation. **c** Sagittal view of the left kidney showing severe dilation of the renal pelvis. **d** Transverse view of the left ureter showing severe dilation1

On physical examination, the dog had a distended abdomen and palpable mid-abdominal mass. Urination resulted in either a weak broken urine stream or infrequent urine dribbling. Hematology and biochemistry yielded no clinically significant abnormalities (creatinine 0.61 mg/dL; reference range, 0.70–1.30 mg/dL). A urine culture was negative for bacterial growth, presumptively due to recent antibiotic administration. Abdominal ultrasonography revealed a 6.2 × 7.8 cm fluid-filled structure consistent with hydronephrosis of the right kidney and severe dilation of the left renal pelvis (1.3 cm; reference range, < 4.0 mm) [14], right ureter (5.5 cm; reference range, < 1.8 mm) [15], and left ureter (1.3 cm; reference range, < 1.8 mm) [15] (Fig. 1a-d). Neither ureter was visualized entering the urinary bladder. A diagnosis of bilateral hydronephrosis and hydroureter, worse on the right was made; however, no cause was identified. Pending availability to perform additional diagnostics, the dog was discharged to return 17 days later. Enrofloxacin was continued at the same dose and frequency as prescribed by the referring veterinarian.

**Fig. 2:**
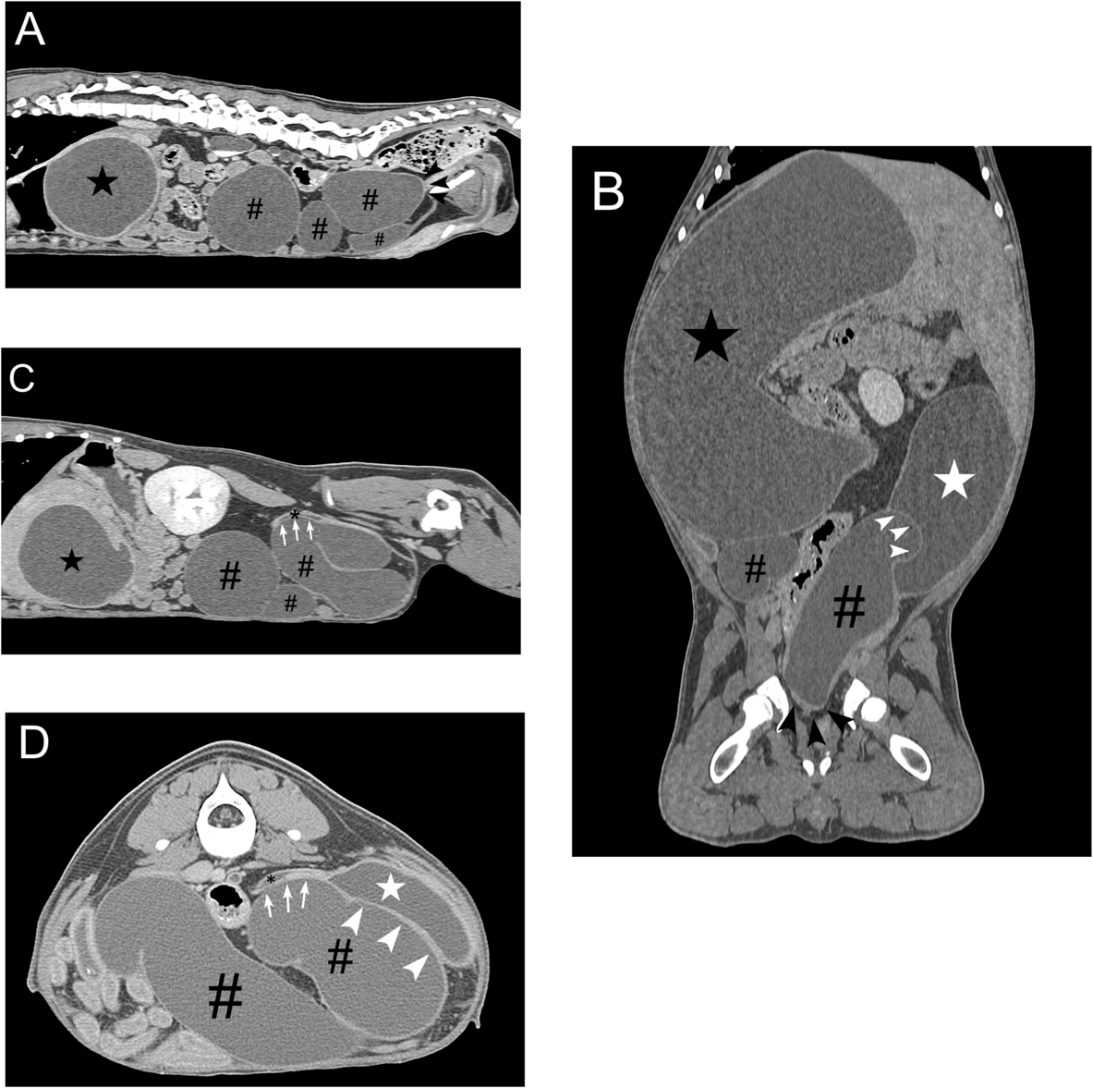
Abdominal computed tomography findings for a dog with distal ureteral atresia. **a** Sagittal view showing blind-ended termination of the right ureter (black arrowhead). Secondary hydronephrosis (black star) and hydroureter (pound sign) is also present. **b** Dorsal view showing compression (white arrowhead) of the urinary bladder (white star) by the dilated atresic ureter (pound sign) and hydronephrotic kidney (black star). The blind-ended termination of the right ureter (black arrowhead) is also present. **c** Sagittal view showing compression (white arrow) of the left distal ureter (black asterisk) by the dilated atresic ureter (pound sign) and hydronephrotic kidney (black star). **d** Transverse view showing compression (white arrowhead and arrow) of the left distal ureter (black asterisk) and urinary bladder (white star) by the dilated atresic ureter (pound sign)

At the follow-up evaluation (visit #2), the dog’s lower urinary tract signs persisted of similar severity to visit #1. A recheck ultrasound of the urinary tract revealed progressive hydronephrosis of the right kidney and progressive dilation of the left renal pelvis, right ureter, and left ureter. Suspecting persistent bilateral obstructive disease, an abdominal computed tomography (CT) scan^2^ was performed. It confirmed similar changes to the kidneys and ureters as demonstrated on ultrasound; however, it additionally identified an abrupt, blind ended termination of the right ureter lacking patency with the urinary bladder and extramural compression of the left distal ureter and urinary bladder by the right, severe hydroureter (Fig. 2a-d). No contrast^3^ was excreted by the right kidney. Following the CT scan, a 3.1 mm, 30° rigid cystoscope^4^ was used to diagnose a left-sided intramural ectopic ureter and an absent right ureteral orifice via a perineal approach as previously described [16]. These findings were consistent with right-sided distal ureteral atresia and hydroureter resulting in extramural compression of the contralateral (left) ureter and urinary bladder. The result of this congenital ureteral abnormality was bilateral hydronephrosis and lower urinary tract signs. Following these procedures, the dog was discharged pending a right-sided ureteronephrectomy. The enrofloxacin, administered for 24 days with no improvement in lower urinary tract signs nor imaging findings, was discontinued.

Twenty-four hours following discharge, the dog re-presented for inability to urinate. Serum creatinine was within reference interval (0.71 mg/dL; reference range, 0.70–1.30 mg/dL). A red rubber urinary catheter was passed into the urethra with ease and 1100 milliliters of serosanguinous urine containing blood clots was removed from the urinary bladder. A fluoroscopic-assisted^5^ retrograde contrast^3^ urethrogram revealed an intraluminal filling defect in the pelvic urethra. Flexible urethroscopy performed immediately thereafter using a 2.8 mm ureteroscope^4^ confirmed mucosal damage and secondary fibrin tags resulting in occlusion of the pelvic urethra, likely iatrogenic from the rigid cystoscopy. An indwelling Foley urinary catheter was placed to allow for urethral healing and clearance of blood clots from the urinary bladder.

**Fig. 3:**
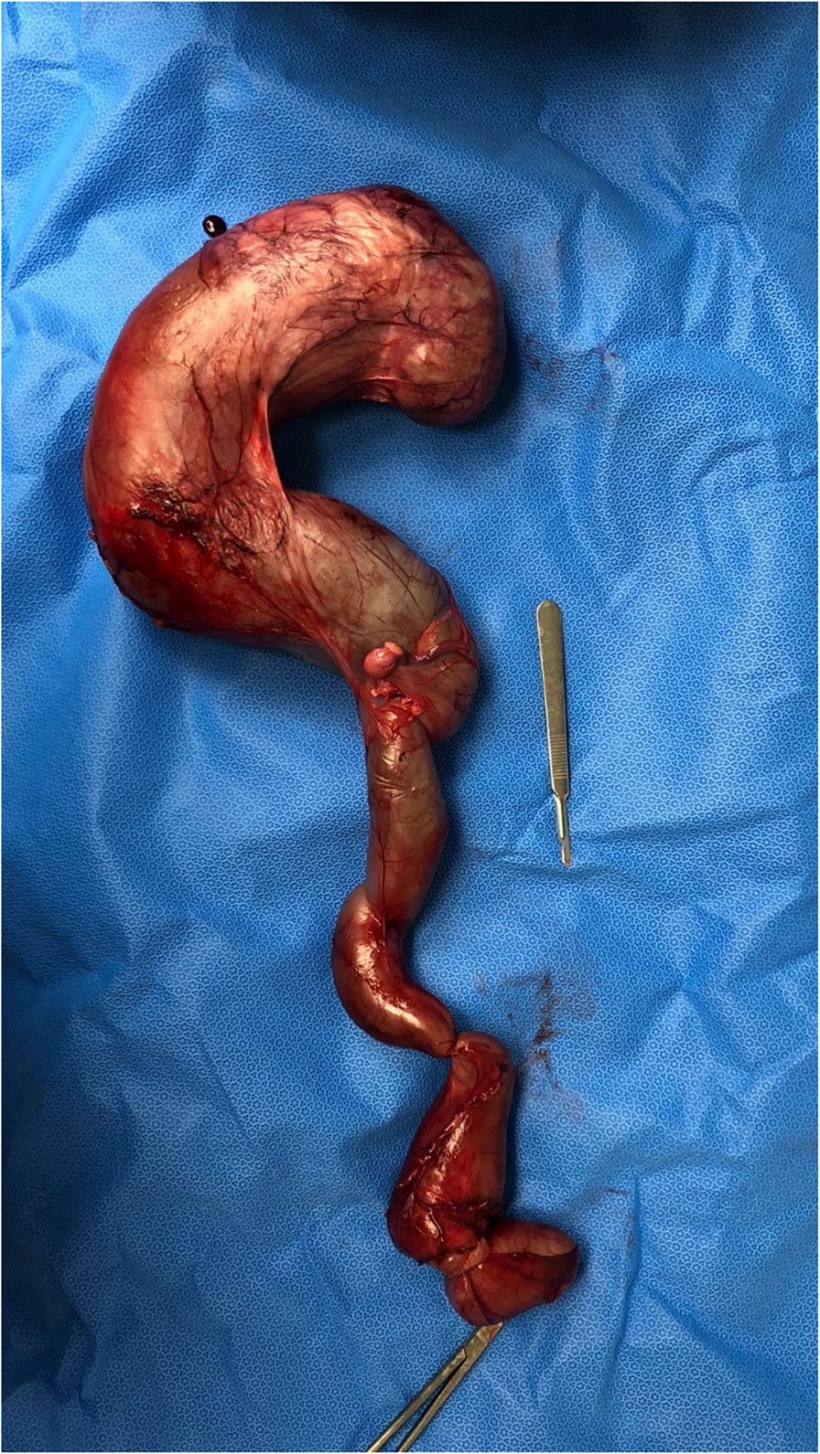
Surgical removal of the right kidney and ureter

Two days later, a right-sided ureteronephrectomy was performed (Fig. 3). Following a fast overnight, the dog was premedicated with midazolam^6^ 0.2 mg/kg IM and hydromorphone^7^ 0.1 mg/kg IM to facilitate intravenous catheter placement. Induction was performed with propofol^8^ 3 mg/kg IV to effect. Following endotracheal intubation, the dog was maintained on isoflurane in 100 % oxygen with a fentanyl^9^ continuous rate infusion (CRI) at 10 µg/kg/hr. and lactated Ringer’s solution at 5 mL/kg/hr. Anesthetic monitoring included electrocardiography, capnography, pulse oximetry, fractional expired isoflurane, fractional inspired oxygen percentage, and direct arterial blood pressure. Several episodes of systemic hypotension occurred, each responding favorably to a 5 mL/kg bolus of IV fluids.

**Fig. 4:**
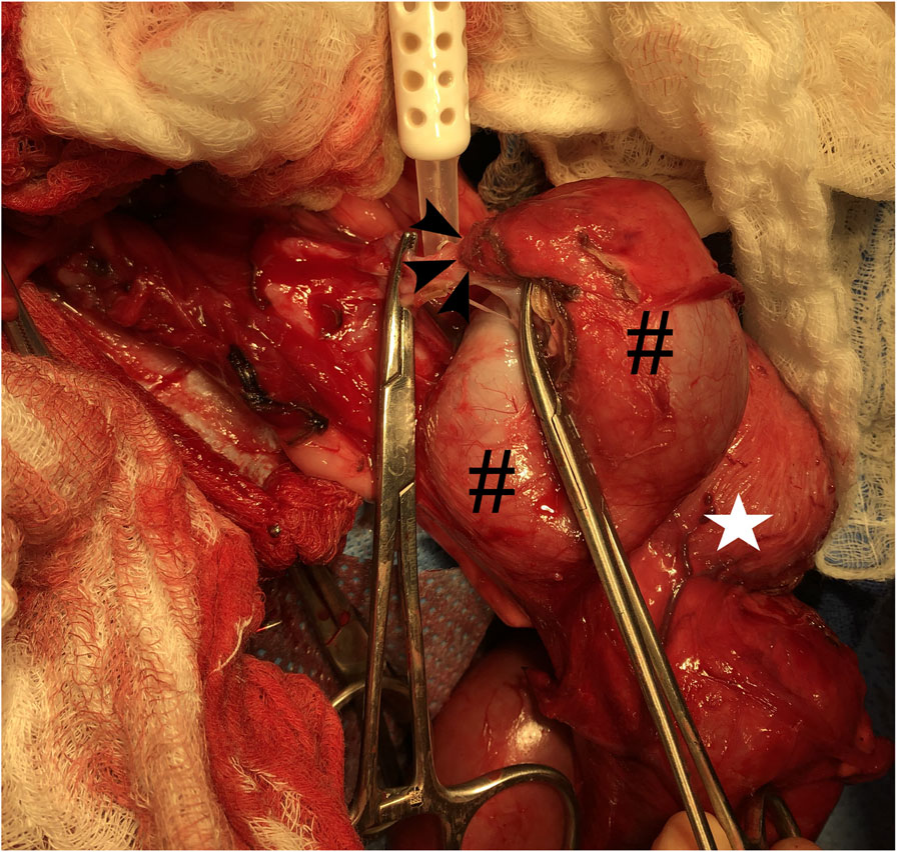
Intraoperative surgical image of the right atresic ureter termination. The blind-ended termination (black arrowhead) of the tortuous right atresic ureter (pound sign) is away from the urinary bladder (white star)

Intraoperatively, the right ureter was severely distended, resulting in extramural compression and displacement of the urinary bladder (Fig. 4). Additionally, there was no patent connection between the right ureter and urinary bladder, as the blind-ended termination of the ureter was attached via fibrous adhesions to the peritoneum (Fig. 4). Removal of the right kidney and ureter occurred without complication. Postoperative management consisted of lactated Ringer’s solution at 60 mL/kg/day and a fentanyl CRI at 3 µg/kg/hr. Twenty-four hours following surgery, the dog was eating well and pain was well-controlled, prompting transition to acetaminophen with codeine^10^ 1 mg/kg PO q8h. The indwelling urinary catheter was left in place for five days following surgery to allow for continued urethral healing. Within six hours following urinary catheter removal, the dog produced a strong, continuous urine stream. No stranguria or pollakiuria were noted. The dog was monitored in-hospital for another twenty-four hours, again during which no stranguria or pollakiuria were reported. The dog was discharged one week after the presentation for urethral obstruction.

Grossly, the right kidney consisted of a thick capsule with no discernable renal tissue and a markedly dilated right ureter that ended in a blind sac. Histologically, the right kidney had scant isolated renal tubules surrounded by haphazardly arranged fibrous tissue and small to moderate numbers of neutrophils and plasma cells. The right ureter had a similar cellular infiltrate as the kidney, with the addition of a diffusely hypertrophied tunica muscularis. The distal blind-ended portion of the ureter contained smooth muscle fibers, confirming ureteral tissue origin. Both gross and microscopic findings were diagnostic of distal ureteral atresia with secondary hydroureter and hydronephrosis. Bacterial cultures of the urine, surgically removed kidney, and fluid within the right hydronephrotic kidney were negative.

**Fig. 5:**
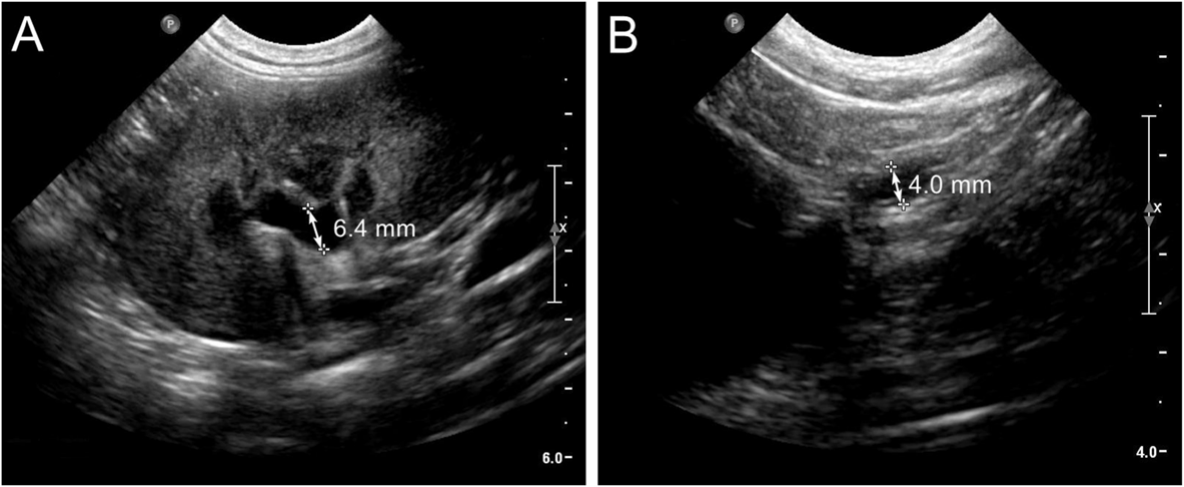
Abdominal ultrasound findings after surgical removal of the right kidney and ureter. **a** Sagittal view of the left kidney showing marked improvement in dilation of the renal pelvis. **b** Transverse view of the left ureter demonstrating a marked reduction in dilation

At a follow-up recheck four months later (visit #3), the dog was urinating a strong urine stream, with no pollakiuria or stranguria noted. Again, serum creatinine remained within reference interval (0.67 mg/dL; reference range, 0.70–1.30 mg/dL). However, the dog was intermittently dribbling urine, likely the result of the left ectopic ureter. Recheck abdominal ultrasound revealed persistent, but vastly improved left hydronephrosis (6.4 mm; reference range, < 4.0 mm) and hydroureter (4.0 mm; reference range, < 1.8 mm) [14, 15] (Fig. 5a-b). Correction of the left ectopic ureter was not attempted because of the mild nature of the incontinence and owner decision. Now nineteen months after surgery, the dog continues to urinate without stranguria and pollakiuria, and serum creatinine remains within reference interval.

## Discussion and conclusions

Ureteral atresia is the congenital absence of a ureteral opening, resulting in a blind-ended ureter that terminates adjacent to the urinary bladder [11]. This anatomical anomaly produces a ureteral obstruction that leads to hydronephrosis, hydroureter, and loss of function of the kidney ipsilateral to the lesion. Ureteral atresia is rare in dogs, as the authors are aware of only one other case being reported [1]. The dog described in that report had right sided hydronephrosis and hydroureter due to distal ureteral atresia with a normal appearing contralateral kidney and ureter. Unique to the dog in this report is obstruction of the contralateral ureter with subsequent hydronephrosis as a result of the dilated atresic ureter. Ureteral obstruction leading to hydronephrosis as a consequence of contralateral hydroureter is also rare in humans [12, 13]. Of clinical importance in the human cases and this report, ureteronephrectomy of the atresic ureter and associated hydronephrotic kidney resulted in a significant reduction of hydronephrosis in the contralateral kidney and preservation of renal function [12, 13].

Like in dogs, ureteral atresia is a rare congenital urinary tract abnormality in humans. It tends to be unilateral, occur most commonly in the distal ureter, be associated with a dysplastic or non-functioning kidney, and diagnosed at a young age [17–22]. Although the exact cause is unclear, ureteral atresia is hypothesized to result from ischemic injury to the developing ureter or a genetic failure of tissue differentiation [23, 24]. During embryological development, the ureter arises from the ureteric bud, the latter of which originates from the mesonephric duct [25]. During ureteral growth, canalization is crucial for the establishment of ureteral patency [25]. Atresia results when this ureteral patency fails to occur, presumably due to one of the aforementioned hypothesized causes. Although the etiology of ureteral atresia in dogs is also uncertain, the numerous similarities between this case and that described in humans would make a common underlying cause plausible [17–22]. In this dog, it is unknown if the right kidney was non-functional due to progressive hydronephrosis or true dysplasia; however, the lack of fetal glomeruli and immature tubules make the latter less likely.

Congenital ureteral anatomic anomalies causing obstructive hydronephrosis and hydroureter are rare in veterinary small animal patients. Reported etiologies include duplication, atresia, stricture, stenosis, circumcaval ureter, and ectopic ureter [1–10]. Classic lower urinary tract clinical signs of hematuria, pollakiuria, and stranguria, when present, were the result of a bacterial lower urinary tract infection [3, 5]. Conversely, the etiology of the lower urinary tract signs in this case was unique, resulting from extramural compression of the urinary bladder by the markedly dilated atresic ureter. The questionable urinary tract infection diagnosis, lack of improvement in clinical signs following extensive (24 days) preoperative antibiotic therapy, negative urine culture during antibiotic treatment, and preoperative ultrasound and CT scan findings provide strong supportive evidence for urinary bladder compression as the cause for the stranguria and pollakiuria reported. Lastly, lower urinary tract clinical signs appear to be uncommon manifestations with any of the congenital urinary tract anatomic abnormalities in small animals [1, 2, 4–9].

The decision to pursue ureteronephrectomy was multifactorial, based primarily on the etiology of the lower urinary tract signs as delineated by CT imaging of the abdomen. Additionally, a lack of discernible renal tissue on ultrasound and contrast uptake following CT indicated negligible function remaining in the right kidney. Lastly, surgical removal of the kidney and ureter is the primary treatment modality in humans with ureteral atresia [17, 19, 21, 22], especially if the hydroureter is causing contralateral ureteral obstruction [12, 13]. The rapid resolution of lower urinary tract signs and substantial reduction in contralateral hydronephrosis indicate that surgical removal was the most appropriate clinical decision. The dog, nineteen months following surgery, continues to have a normal serum creatinine value and be lower urinary tract sign free.

In addition to ureteral atresia, a left-sided ectopic ureter was present and likely accounted for the urine dribbling in this dog. Though there was a significant reduction of the left hydroureter and hydronephrosis following surgery, the lack of resolution is explained by the uncorrected ectopic ureter [8, 9]. Ideally, correction of this ectopic ureter would have been pursued; however, owner election against this prevented it from occurring. Furthermore, the dog’s urinary incontinence was very mild.

We acknowledge several potential limitations of this case report. It can be argued that the dog’s lower urinary tract signs were the result of a urinary tract infection and that the upper urinary tract manifestations of hydroureter and hydronephrosis improved on the visit #3 imaging as a result of treatment for an ascending bacterial infection (pyelonephritis). Although possible, we feel these explanations for the findings that make this case report novel are less likely for several reasons. First, the urinary tract infection diagnosis is unconvincing, as it is based solely on the presence of leukocytes found on a free-catch urine dipstick sample. Second, the lower urinary tract signs did not improve despite a duration (24 days) of antibiotic therapy that would be considered sufficient to see improvement in clinical signs. Additionally, the negative urine culture one week into the antibiotic treatment indicated that the enrofloxacin therapy was adequate to sterilize the urine, thus appropriately treating any potential bacterial infection present. Third, dilation of the contralateral (left) kidney and ureter worsened on the visit #2 preoperative ultrasound images following 24 days of antibiotic treatment. Finally, the dog displayed no systemic signs (i.e. lethargy, fever, anorexia) nor had blood work abnormalities (i.e. inflammatory leukogram, renal azotemia) consistent with an ascending infection. In totality, although we cannot exclude the possibility of a concurrent urinary tract infection, these reasons make an infection involving both the lower and upper urinary tract an unlikely contributor to the unique manifestations occurring secondary to the atresic right ureter in this case report.

This is the first case report in any animal species of a congenital ureteral anatomic abnormality causing lower urinary tract signs and contralateral ureteral obstruction and hydronephrosis due to extramural compression of the contralateral ureter and urinary bladder. This report expands our understanding of congenital lower urinary tract disease in dogs; albeit rare, this abnormality is an important differential diagnosis in a young dog with bilateral hydronephrosis and lower urinary tract signs, especially if a bacterial urinary tract infection has been ruled out. Furthermore, this case demonstrates that ureteronephrectomy represents a viable long-term treatment option, despite contralateral hydronephrosis being a sequalae of the original anatomic abnormality. Lastly, the diagnostic workup in this case illustrates how CT is becoming incrementally more important for in the diagnosis and treatment of some congenital urogenital diseases.

## Data Availability

All data generated or analyzed during this study are included in this published article.

## Abbreviations

PO: Per os
CT: Computed tomography
IM: Intramuscular
IV: Intravenous
CRI: Continuous rate infusion

## Footnotes

1 Baytril^®^, Bayer Healthcare, Shawnee Mission, Kansas

2 Toshiba Aquilon 16, Toshiba American Medical Systems, Tustin, California

3 Omnipaque 300 mgI/mL, GE Healthcare, Marlborough, Massachusetts

4 Karl Storz Endoscopy-America, El Segundo, California

5 CombiDiagnost R90, Philips Medical Systems, Nashville, Tennessee

6 Versed^®^, West-Ward Pharmaceutical, Eatontown, New Jersey

7 Dilaudid^®^, Hospira, Lake Forest, Illinois

8 Diprivan^®^, Zoetis, Kalamazoo, Michigan

9 Sublimaze^®^, Hospira, Lake Forest, Illinois

10 Tylenol^®^ with Codeine 3, Janssen Pharmaceutical, Titusville, New Jersey
